# Guy's Hospital

**Published:** 1905-04-01

**Authors:** H. Cosmo Bonsor

**Affiliations:** Treasurer


### GUY'S HOSPITAL.

By Mr. H. Cosmo Bonsor, Treasurer.

I shall be grateful for the publication in your valuable columns of the enclosed appeal from the great hospital of which I am treasurer, and for leave to add a few words to commend it to your readers.

In the first place it should be understood that this is not a fresh demand, but an appeal for the balance of the funds sought to be raised in 1901. This balance, while of comparativaly small amount, is one upon which the well being and stability of the institution depend.

It may be remembered that in 1901 Guy's Hospital appealed for £180,000 to cover the cost of renovations, buildings, and equipments, which had become indispensable to the efficiency of this old institution, and necessary to the reopening of closed wards encouraged by King Edward's Hospital Fund for London. The works have been carried out, but only part of the money has been forthcoming, and we need at once £100,000 with which to discharge pressing liabilities.

Secondly, Guy's at that date appealed, and now repeats the appeal, for an increase in its regular income. The cost of maintenance, since all the wards are opened and more than 600 beds available for patients, is not at any time likely to be less than £62,000 per annum, to which must be added a moderate estimate of £4,000 per annum for extraordinary expenditure. Towards the total of £66,000 per annum the hospital possesses an income, more or less assured, of some £51,000 per annum. This includes income from endowments, from King Edward's Hospital Fund for London, the Hospital Saturday and Sunday Funds, and from annual subscribers. For the balance of £15,000 per annum the hospital is at the mercy of chance support, and of late the deficit has had to be met by way of loans.

Such a position cannot be regarded as satisfactory. The pride which all should feel in the possession of this splendid heritage of eighteenth-century philanthropy might well be expected to produce a supply sufficient for every need. But, historical sentiment apart, the Governors are content for the case to be judged on the vast service to humanity which Guy's Hospital is rendering to-day. In 1904 its in-patients numbered 8,581, and no less than 136,872 sufferers, most of whom came from the poorest parts of South London, were treated in the Out-Patient Departments. The need of these poor people is genuine; it is increasing year by year, and it must be met. The hospital is ever ready and willing to do the work if kind hearts will provide the means.

And I cannot doubt that they will, remembering, as I do, with deep gratitude the noble support which Guy's Hospital has received since the year 1895, when the Governors put their hands to the heavy double task of bringing this institution abreast of modern requirements and of repairing the disaster which had overtaken its endowments.

Let me, in conclusion, make quite clear two things of special significance at the present time. Guy's Hospital restricts its ministrations to fit recipients, and every penny entrusted to us for the relief of the sick poor goes to their direct benefit.

